# Therapeutic Targets in Innate Immunity to Tackle Alzheimer’s Disease

**DOI:** 10.3390/cells13171426

**Published:** 2024-08-26

**Authors:** Maria L. Serradas, Yingying Ding, Paula V. Martorell, Ida Kulińska, Sergio Castro-Gomez

**Affiliations:** 1 Institute of Physiology II, University Hospital Bonn, 53115 Bonn, Germany; 2 Institute of Clinical Chemistry and Clinical Pharmacology, University Hospital Bonn, 53127 Bonn, Germany; 3 German Center for Neurodegenerative Diseases (DZNE), 53127 Bonn, Germany; 4 Center for Neurology, Department of Parkinson, Sleep and Movement Disorders, University Hospital Bonn, 53127 Bonn, Germany

**Keywords:** Alzheimer’s disease, innate immunity, neuroinflammation, therapeutic targets

## Abstract

There is an urgent need for effective disease-modifying therapeutic interventions for Alzheimer’s disease (AD)—the most prevalent cause of dementia with a profound socioeconomic burden. Most clinical trials targeting the classical hallmarks of this disease—β-amyloid plaques and neurofibrillary tangles—failed, showed discrete clinical effects, or were accompanied by concerning side effects. There has been an ongoing search for novel therapeutic targets. Neuroinflammation, now widely recognized as a hallmark of all neurodegenerative diseases, has been proven to be a major contributor to AD pathology. Here, we summarize the role of neuroinflammation in the pathogenesis and progression of AD and discuss potential targets such as microglia, TREM2, the complement system, inflammasomes, and cytosolic DNA sensors. We also present an overview of ongoing studies targeting specific innate immune system components, highlighting the progress in this field of drug research while bringing attention to the delicate nature of innate immune modulations in AD.

## 1. Introduction

Due to advances in healthcare and increased longevity, societies of all industrialized and non-industrialized countries are seeing a significant increase in the prevalence of common neurodegenerative disorders such as Alzheimer’s disease (AD). These devastating disorders represent the biggest challenge and an immense economic burden for all health systems around the world. AD is the most common neurodegenerative disorder characterized by a progressive neuronal loss that leads to a decline in memory and other cognitive functions, i.e., dementia, culminating in the loss of independence in activities of daily living. To illustrate the potential magnitude of AD’s impact, epidemiologists have estimated that the global prevalence of AD in people older than 65 years will rise to 150 million by 2050 [1]. This alarming estimation shows the urgency to develop novel strategies for the prevention and treatment of this gigantic public health challenge.

Histologically, AD is defined by two core neuropathological hallmarks: the accumulation of extracellular amyloid β (Aβ) plaques and intracellular neurofibrillary tangles (NFTs) formed by misfolded and hyperphosphorylated tau (p-tau) protein [2]. However, classical neuropathological features begin even decades before the first cognitive symptoms manifest [3]. Additionally, the pre-pathological stages of AD are thought to be characterized by latent neuroinflammation [4], alterations in the blood–brain barrier [5], accelerated cellular senescence [6], and dysfunction of the glymphatic system [7]. This cellular phase occurs alongside the processing and accumulation of Aβ aggregates. According to the current but debated hypothesis, Aβ triggers tau pathology. The neuroanatomical spread of misfolded tau species correlates tightly with the clinical progression of the disease [8]. 

Innate immune activation and subsequent neuroinflammation occur early in the cellular phase and are present throughout the AD continuum. These processes are considered to be essential in the initiation and progression of the disease [9]. Microglia, the main immune cells of the brain, are found to be activated in humans [10] and animal models [11,12] even before the deposition of Aβ plaques and the spread of tau pathology in cortical networks. These observations together with a body of scientific studies summarized in this review clearly show that early preclinical innate activation and neuroinflammatory responses from microglia and other cells must be investigated in more detail to develop effective preventive and disease-modifying therapies. 

**Figure 1:**
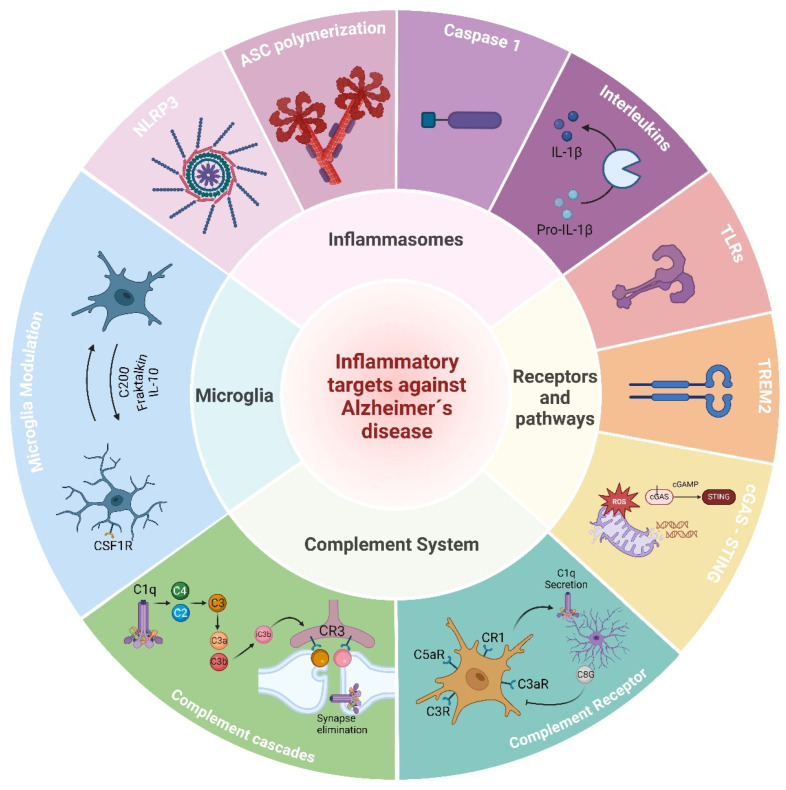
**Emerging immune targets in Alzheimer’s disease (AD).** Emerging immune targets against AD highlight key molecular pathways and molecules implicated in neuroinflammation and immune dysregulation. Chronic neuroinflammation, driven by glial cell activation and the release of cytokines and chemokines, exerts detrimental effects on the CNS. Contrary to earlier beliefs that brain inflammation was merely a passive response to neuronal loss, current research underscores that neuroinflammation significantly contributes to AD progression, potentially even more than amyloid plaques and neurofibrillary tangles. The most promising inflammatory targets identified include microglia modulation, activation/inhibition of inflammation-related receptors and pathways, targeting inflammasome components, and modulation of the complement system. These targets represent potential therapeutic avenues to mitigate AD progression by addressing the underlying inflammatory processes. The figure was created with BioRender.com, accessed on 18 August 2025.

**Figure 2:**
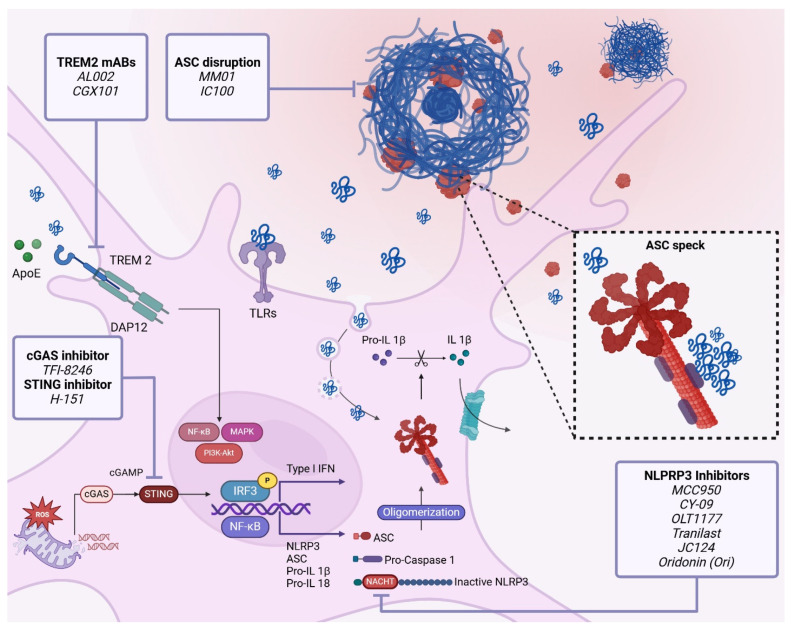
**Current targets on key neuroinflammatory molecules and pathways in AD.** Several treatments are in pre-clinical and clinical trials, aiming to mitigate neuroinflammation as AD treatment. Among the most studied are inflammasome cascade inhibitors, particularly NLRP3 inhibitors, and molecules disrupting the ASC complex, which have shown promise in reducing amyloid-beta plaque formation and enhancing cognition. Additionally, antibodies targeting TREM2 to promote its activation and inhibitors of the STING pathway are being tested, with both approaches demonstrating improvements in AD pathology. These innovative strategies highlight the potential of targeting neuroinflammation to combat AD progression. The figure was created with BioRender.com, accessed on 18 August 2025.

In the following review, we summarized the role of microglia as the main actors in the innate immune responses of the brain and the pathogenesis of AD. We discuss several pathways related to innate activation in AD and potential targets such as microglia, TREM2, the complement system, inflammasomes, and cytosolic DNA sensors (Figure 1). We highlight novel promising compounds and interventions that are being explored experimentally and are paving the way in translational medicine and future clinical studies (Figure 2).

## 2. Current Therapies: Limitations of Past and Current Strategies

Physicians and healthcare providers are still treating AD patients merely symptomatically. Anti-muscarinic compounds (rivastigmine, donepezil, and galantamine) alone or in combination with the N-Methyl-D-aspartic acid (NMDA) receptor modulator memantine were for years the only approved supportive pharmacological therapies for mild-to-moderate dementia due to AD, showing a minimal effect in the progression of cognitive decline or delay for nursing home care [13]. With this limited drug repertoire, the research and development of AD therapies focused for decades on targeting classical neuropathological hallmarks, mainly Aβ processing. However, discouragingly for the entire field, all clinical trials with small molecules targeting the amyloid cascade (e.g., BACE1 inhibitors) or active immunization against Aβ failed to reach any significant clinical endpoint. Several of these clinical trials were even stopped due to worsening cognitive outcomes or serious side effects [14]. These disappointing results have led to questioning the amyloid hypothesis and rethinking clinical trial design as well as diagnostic methods. 

The very recent approval by the USA Food and Drug Administration (FDA) of two monoclonal antibodies against Aβ for passive immunotherapy (lecanemab and donanemab) has raised some hope for an era of disease-modifying therapies. These molecules showed a high efficacy in removing Aβ detected with Positron emission tomography (PET) tracers. However, this passive immune therapy shows only marginal but statistically significant slowing in some clinical parameters that measure independence in daily life activities and cognitive deterioration when the treatment is initiated in patients with mild cognitive impairment (MCI) or mild dementia [15,16]. These results suggest that anti-Aβ therapies must be begun in the asymptomatic and pre-pathological phases of the disease or that Aβ may contribute only partly to AD pathogenesis. Additionally, amyloid-related imaging abnormalities (ARIA) of edema and effusions are detected in more than 20% of patients receiving these immunotherapies, especially those carrying the *APOE4* gene variants. These inflammatory changes are triggered by the targeted Aβ aggregates on the blood vessel walls and lead to an increased risk of serious adverse events such as cerebral bleeding [17]. Furthermore, the estimated cost of these therapies, including follow-up diagnostic workups, is calculated at more than 20,000 USD yearly per treated patient. This elevated bill represents insufficient cost-effectiveness for health systems and has halted the approval by the European Union and the United Kingdom [18]. 

The current panorama of disease-modifying therapies in AD is incipient but optimistic. Prospective long-term studies in pre-symptomatic patients are still necessary to determine whether anti-Aβ passive immunotherapies are effective as a feasible preventive option. Additionally, novel targets based on different hypotheses must be explored, including the inflammatory hypothesis of AD.

## 3. Neuroinflammation as a Central Mechanism in AD

Over the last few decades, several investigations have demonstrated that in addition to Aβ plaques and NFTs, the brains of AD patients exhibit sustained low-grade inflammatory responses even in the pre-pathological phases of the disease [19]. Inflammation is a crucial defense mechanism of the brain against infections, toxins, and injuries. However, a sustained imbalance between anti- and pro-inflammatory signals can lead to chronic neuroinflammation, characterized by the activation of glial cells and the release of cytokines and chemokines, that when not ceased, have detrimental effects on the CNS. In the context of AD, it was initially believed that sustained inflammation in the brain was merely a passive response or consequence triggered by the neuronal loss characteristic of the disease, together with the emergence of Aβ plaques and NFTs. However, contrary to previous assumptions, numerous studies now emphasize that neuroinflammation in AD contributes significantly, possibly even more than the plaques and tangles themselves, to the disease’s progression [19,20].

The notion that inflammation might contribute to AD pathology was first proposed by a pioneering study by McGeer et al. in 1993 [21]. The findings from a small, placebo-controlled, double-blind trial investigating the nonsteroidal anti-inflammatory drug (NSAID) indomethacin in AD patients suggested that NSAID intake slowed cognitive deterioration over the six-month trial period, whereas the placebo group showed a decline as expected. This trial brought to light two significant observations: the presence of reactive microglia in AD brains, suggesting chronic inflammation, and the observation that individuals with rheumatoid arthritis, who frequently use NSAIDs over long periods, had a reduced risk of developing AD [21,22]. Subsequently, in 2000, Akiyama et al. proposed in their review “The Inflammatory Hypothesis of Alzheimer’s Disease”, which provided a comprehensive revision of the role of inflammation in AD, that chronic neuroinflammation contributes significantly to the pathogenesis and progression of the disease [23]. Since then, a growing body of literature has underscored the significance of neuroinflammation as one of the central cellular and molecular mechanisms that drive disease initiation and progression, shedding light on promising avenues for future research and therapeutic interventions.

This pivotal and independent role of the immune system and neuroinflammation is supported by the extensive and reproducible results from multiple genome-wide association studies (GWASs), as more than half of enrichments in AD-risk genes are found in this category or in pathways mainly related to microglia/myeloid function. Furthermore, rare AD risk variants (i.e., *TREM2*, *CD33*, *ABCA7*, *PLCG2*, and *ABI3*) have been found in genes mainly expressed in myeloid cells [24]. Moreover, all non-genetic risk factors for developing AD are to some extent related to neuroinflammatory responses. For example, aging is a low-grade chronic inflammatory condition and the most important environmental risk factor for AD [25]. Mild-to-moderate traumatic brain injury (TBI) comes in second place as the most important non-genetic, non-aged-related risk factor for developing dementia and AD. Secondary damage after TBI is characterized by sustained neuroinflammation and the aggregation of misfolded proteins that may trigger AD and other neurodegenerative disorders [26]. Other inflammatory conditions associated with a higher risk of developing AD are systemic infections [27,28] and obesity in middle age [29]. Additionally, immune-related proteins and cells, such as microglia and inflammasome complexes, have been found in close proximity to AD neuropathological hallmarks such as Aβ plaques, further highlighting the involvement of neuroinflammation in AD pathogenesis [20].

## 4. Targeting Inflammation and the Immune System in AD

Research into novel immune targets against Alzheimer’s disease (AD) continues to evolve, with a focus on identifying the key cellular and molecular pathways involved in neuroinflammation and immune dysregulation. The emerging immune targets being investigated for AD include the following.

### 4.1. Microglia

Microglia, as the main resident immune cells of the CNS, play a pivotal role in innate immunity and brain inflammation, as well as within the context of AD [10,24,30,31]. Under normal conditions, microglia remain in a “homeostatic state”, characterized by ramified morphology with small somas. In this state, they continually survey the environment, communicating with neurons and other glial cells through various signaling mechanisms. Upon detecting threats such as the invasion of pathogens, injury, or pathological protein aggregates, microglia undergo activation, marked by morphological changes: the retraction of processes, enlargement of the cell, and eventually proliferation and migration to the lesion site, where they initiate an innate immune response [32]. The detection of pathological triggers is mediated by pattern recognition receptors (PRRs), encompassing both pathogen-associated molecular patterns (PAMPs) from pathogens and damage-associated molecular patterns (DAMPs) from stressed or dying cells. DAMPs include various molecules like stress-induced proteins, mitochondrial components, nuclear proteins, and DNA. Upon ligand recognition, PRRs and other DAMP-sensing receptors initiate intracellular signaling pathways, culminating in the transcription and release of innate immune mediators such as complement factors, cytokines, chemokines, and growth factors [4]. PRRs are widely expressed by various cell types in the central nervous system, including microglia, astrocytes, oligodendrocytes, endothelial cells, and even neurons to some extent. These receptors comprise different families, including toll-like receptors (TLRs), C-type lectin-like receptors (CLRs), nucleotide-binding domain with leucine-rich repeat-containing receptors (NOD-like receptors or NLRs), retinoic acid-inducible gene-like receptors (RLRs), absent in melanoma 2 protein-like receptors (ALRs), and cytosolic DNA receptors (CDSs). While TLRs and CLRs are membrane-bound, the others are cytoplasmic receptors [4].

#### 4.1.1. The Role of Microglia in Alzheimer’s Disease

In AD, microglial activation is hypothesized to be primarily driven by the presence of Aβ oligomers and fibrils decades before the formation of Aβ plaques. These oligomeric forms are recognized by PRRs and other DAMP-sensing receptors, including RAGE, CD36, CD14, CD47, and toll-like receptors (TLR2, TLR4, TLR6, and TLR9) [33,34]. While activated microglia initially phagocytose Aβ, prolonged activation may lead to their enlargement, impaired Aβ processing, and plaque formation. Early in AD pathogenesis, the immune response can facilitate Aβ clearance, showing positive effects on AD-related pathologies in animal models. However, sustained activation of the immune response, characterized by microgliosis and pro-inflammatory signaling, can exacerbate AD pathology and neural death [20,35]. The depletion of microglia in an Aβ-amyloidosis model (5xFAD) induces a profound shift from parenchymal amyloid plaques to cerebral amyloid angiopathy (CAA) [36,37]. Mice whose microglia were chronically depleted presented accelerated mortality, brain calcifications, a decline in white matter integrity, and neurodegeneration in aging [38]. These results indicate that microglia are essential in keeping the integrity of brain networks during aging and actively participate in the aggregation and formation of Aβ-amyloid plaques by mechanisms that still need deeper investigation.

#### 4.1.2. Microglia Modulators against Alzheimer’s Disease

The survival and proliferation of microglia during development and in the adult brain are dependent on colony stimulating factor 1 receptor (CSF1R), whose cognate ligands are CSF-1 and IL-34. CSF1R can be inhibited by several compounds already tested in preclinical settings, such as PLX3397 (pexidartinib) [39], PLX5622 [40], BLZ945 [41], Ki20227 [42], JNJ-527 [43], ARRY-382 [44], and GW2580 [45]. Although the chronic and complete depletion of microglia using these inhibitors can bring the aforementioned deleterious side effects, the transient and partial depletion of microglia in several models of AD seems to exert beneficial outcomes. Eliminating 50 to 80% of microglia in 5xFAD mice rescues dendritic spine loss, reduces overall inflammation, and improves cognitive deficits [36,46,47,48]. Early treatment of 6-month-old APP/PS1 mice with GW2580 for 3 months inhibits normal microglia proliferation with an overall reduction of 30% and prevents synaptic degeneration and behavioral deficits without affecting amyloid levels [49]. Similar effects can be achieved in transgenic models expressing frontotemporal dementia (FTD)-associated *MAPT* mutations. Eight-month-old P30IS mice treated with JNJ-527 show a reduction in microglia of approximately 40% with a subsequent decreased expression of pro-inflammatory cytokines, reduced neuronal death, and rescued motor neuron degeneration [43]. The use of PLX5622 in the 3xTg-AD model with a reduction in microglia of approximately 30% has also been shown to prevent microglial association with plaques and improve hippocampal-dependent memory deficits [40]. Furthermore, stimulating microglia proliferation by injecting CSF1 weekly in the APPSwe model prior to cognitive impairment prevents further memory deficits and decreases Aβ deposits [50]. The modulation of microglia proliferation and survival by targeting CSF1R not only demonstrates the essential role of these cells in pre-pathological stages of AD but also proves that eliminating “senescent” microglia or stimulating the proliferation of new homeostatic microglia may be a feasible strategy to prevent AD pathology. 

During physiological conditions, microglia are constantly surveilling neuronal networks and brain structure stability through the sensing of neuronal milieu and by establishing transient but constant physical contact with synaptic structures, blood vessels, and other brain cells [51]. The mechanisms by which microglia homeostasis is induced or maintained remain underexplored. IL-10, Cluster of Differentiation 200 (CD200 or OX-2 membrane glycoprotein), and CX3CL1 (fractalkine) are some of the proposed main molecules that prevent microglia hyperactivation under homeostatic conditions. 

IL-10 is a homodimeric polypeptide of 17 kD, usually produced by immune cells (T cells, B cells, macrophages, and other hematopoietic cells) after their activation. The IL-10 signal is transduced through a dedicated receptor (IL-10Ra) and an accessory co-receptor (ILa-10Rb) which leads to the JAK/STAT pathway and dampens cytokine gene expression and antigen presentation [52]. In vitro, IL-10 decreases the proinflammatory activity of microglia and other glial cells [53]. Similarly, the activation of the IL-10R on microglia prevents hyperactivation in a model of septicemia [54]. Moreover, *Il10* gene deficiency potentiates LPS-induced tau pathology in non-transgenic mice [55]. In a counteractive manner, the constitutional removal of the *il10* gene ameliorates phagocytic deficits of microglia and mitigates Aβ-amyloidosis, CAA, and cognitive deficits in APP/PS1 mice [56]. The viral transduction of IL-10 in the brain of AD mouse models has resulted in conflicting outcomes. While the intrahippocampal application of adenovirus-associated virus vectors expressing IL-10 (AAV–IL-10) in transgenic APP mice (Tg2776) exacerbates Aβ plaque burden and memory deficits [57], the injection of a similar vector mediating IL-10 overexpression in the hippocampi of APP-PS1 mice showed enhanced neurogenesis and an improvement in spatial learning deficits [58]. Although conflicting, the targeting of IL-10 pathways may have therapeutic consequences for AD. Further studies are necessary to determine the critical window and duration for the treatment based on this target. 

CD200 is a type I transmembrane glycoprotein member of the immunoglobulin superfamily that is commonly expressed in T cells, B cells, dendritic cells, and neurons [59]. The expression of the CD200 receptor (CD200R) is mainly found in myeloid cells (microglia, macrophages, and dendritic cells), but also in astrocytes and oligodendrocytes. The CD200–CD200R interaction exerts an inhibitory effect on microglia, likely by triggering the intracellular phosphorylation of Dok1 and Dok2 and the further inhibition of the Ras-PI3K and Ras-ERK pathways, as well as the subsequent inhibition of pro-inflammatory signals through the downregulation of NF-κB [60]. In *Cd200^−/−^* mice, the microglia exhibited a hyperactivated phenotype, and injured *Cd200^−/−^* neurons elicited accelerated microglial responses [61]. In AD patients, CD200 and CD200R are found to be downregulated in postmortem brain tissue [62], and the viral expression of CD200 in the hippocampi of APP/PS1 mice improves cognitive functions by promoting synaptic plasticity [63]. These preliminary results encourage the development of future trials based on the modulation of the CD200–CD200R pathway.

Another microglia modulator extensively studied over the last few decades is the chemokine fractalkine or C-X_3_-C motif ligand 1 (CX_3_Cl1), the expression of which is abundant in neurons [64,65]. In homeostatic conditions, CX_3_Cl1 is found as a membrane-anchored protein, but under inflammatory challenges, it is cleaved by metalloproteases such as disintegrin and metalloproteinase domain-containing protein 10 (ADAM10) and ADAM17 [66]. The anchored and soluble CX_3_Cl1 (sCX_3_Cl1) binds to a unique G protein-coupled receptor (CX_3_CR1) that activates several pathways, leading to increased calcium concentrations in the microglia cells. The fractalkine/CX_3_CR1 axis plays an essential role in CNS development [67,68] and the physiological maintenance of neuronal network stability and synaptic plasticity in the adult brain [69,70]. It also exerts modulatory effects on microglia activity, acting as an anti-inflammatory during acute neuroinflammation [71,72,73,74]. In AD patients, CX_3_Cl1 is found to be upregulated in postmortem brain tissue of early pathology stages, especially in the cortex, and correlates with the progression of tau pathology [72]. Similarly, sCX_3_Cl1 increases in the CSF and blood of patients with mild cognitive impairment due to AD [75]. The role of the fractalkine/CX_3_CR1 axis in AD is still under debate since *cx3cr1* knockout (*cx3cr1^−/−^*) has shown opposite effects in amyloidosis and tau pathology models. While *cx3cr1^−/−^* decreases Aβ plaque burden in APPPS1, R1.40 [76], and APP/PS1 mouse models [77], the constitutional *cx3cr1* deficiency worsens tau pathology in the hTau model [74,78] and accelerates the spread of tau aggregates [79]. Additionally, the AAV-mediated overexpression of sCX_3_Cl1 in the hippocampi of rTg4510 mice slows tau pathology and neurodegeneration without having any behavioral effect [80]. In 3xTg-AD mice, a combined model of β-amyloidosis and tauopathy, the constitutive lack of the *cx3cr1* gene prevents neuronal loss [81]. 

Several pharmacological modulators of the fractalkine/CX_3_CR1 axis have been developed in recent years. One of the most promising is the small allosteric CX_3_CR1 antagonist AZD8797 [82], whose neuroprotective effect has been demonstrated in rat models of chronic-relapsing multiple sclerosis [83] and acute spinal cord injury [84]. Additionally, the synthetic peptide Tet34 derived from the CX3CL1 intracellular domain [85] and E6011, an inhibitory monoclonal antibody against CX3CL1, are being investigated in preclinical and clinical studies of peripheral inflammatory diseases. E6011 has shown a long-term safety profile in randomized clinical trials for rheumatoid arthritis [86]. These novel treatments remain to be tested in preclinical and clinical AD studies and may represent an attractive option for future research. 

### 4.2. Triggering Receptor Expressed on Myeloid Cells 2 (TREM2)

Triggering receptors expressed on myeloid cells (TREMs) constitute a family of cell-surface receptors found widely across myeloid cells. Initially, the discovery of TREM1 unveiled its role as an immune response amplifier, particularly in enhancing granulocyte and monocyte reactions to microbial stimuli. Since then, the TREM family has expanded to encompass proteins expressed not only on granulocytes and monocytes but also on macrophages and dendritic cell (DC) lines. Broadly, TREMs serve as modulators, dictating the threshold and duration of myeloid cell responses [87]. One prominent member of this family, TREM2, which operates as an activating receptor found on tissue macrophages, both on their surface and within intracellular reservoirs, has received great attention in the context of AD. TREM2-positive macrophages include microglia within the central nervous system, osteoclasts in bone tissue, and various macrophage subsets distributed in the liver, adipose tissue, skin, gut, and tumors. TREM2 binds to microbial lipids such as bacterial lipopolysaccharides (LPSs) and non-glycosylated mycolic acids found in mycobacteria. Additionally, TREM2 has been reported to engage with proteins prone to aggregation and accumulation in neurodegenerative disorders. Notably, TREM2 has an affinity for Aβ peptides [88]. This multifaceted binding profile underscores TREM2’s pivotal role in orchestrating immune responses, particularly in the context of neuroinflammation and neurodegenerative diseases like AD [87,89]. 

The signaling pathway of TREM2 in the brain involves the initial binding of TREM2 to ligands, such as phosphatidylserine, phosphatidylethanolamine [90], low-density lipoprotein, apolipoprotein E (ApoE) [91], or Aβ peptides [92], which triggers conformational changes and facilitates its interaction with adaptor proteins like the DNAX activation protein of 12kDa (DAP12). This interaction leads to the phosphorylation of immunoreceptor tyrosine-based activation (ITAM) motifs within DAP12 by Src family kinases. Phosphorylated ITAMs serve as docking sites for cytoplasmic signaling molecules, including Syk kinase. Syk kinase activation triggers downstream signaling cascades, leading to the activation of various intracellular pathways such as the PI3K-Akt pathway, MAPK pathway, and NF-κB pathway. The activation of these signaling pathways culminates in gene expression programs and cellular responses such as cytokine production, enhanced phagocytosis, and the regulation of inflammatory responses. Tight regulation by negative feedback mechanisms ensures the proper control of TREM2 signaling to prevent excessive or dysregulated immune responses, underscoring its critical role in maintaining brain homeostasis and health [4,87,89].

#### 4.2.1. Role of TREM2 in Alzheimer’s Disease Pathogenesis and Progression

In AD, TREM2 has garnered significant attention due to its critical role in the brain’s immune response and its potential involvement in clearing Aβ plaques. In fact, TREM2 has been shown to be involved in Aβ pathology regulation, the hyperphosphorylation of tau protein, and microglia activation [88]. More than 60 different genetic variants have been identified in the *TREM2* gene and linked to several neurological disorders, but the most common are linked to an increased risk of developing AD [93,94,95]. The most notable *TREM2* mutation variant is the R47H (Arg47His), characterized by a substitution of histidine for arginine in position 47 of the TREM2 protein. This variant has impaired binding capabilities to cells, ApoE, and damage-associated lipids or apolipoprotein ligands [96]. Additionally, R47H may confer a loss of TREM2 function, leading to a reduced clearance of Aβ peptides and other toxic substances. Epidemiological studies have shown that the R47H variant is enriched in AD cases and less prevalent in cognitively normal controls. The R47H variant may contribute to an earlier age of onset in AD, in addition to an increased risk of AD. Another prominent *TREM2* mutation that disrupts its binding capabilities to cells and ApoE, but not to lipid ligands, is the R62H (Arg62His) variant. Epidemiological studies have shown a mild increased risk of AD in individuals harboring the R62H variant. Another variant, the H157Y (His157Tyr), has also been associated with AD. This mutation is located in the stalk domain of TREM2, specifically, in the cleavage site of metalloproteases ADAM, resulting in the increased shredding of TREM2 and reduced cell surface expression. This variant has a deleterious and damaging impact on the functions of TREM2 and significantly increases AD susceptibility in carriers, although it is rarely found in AD patients. Other *TREM2* mutations that have been associated with AD, although less common in the general populations, are D87N (Asp87Asn), T96K (Thr96Lys), L211P (Leu211Pro), and R136Q (Arg13Gln) [97]. 

The role of TREM2 in AD pathophysiology is currently not fully understood. In studies conducted by Wang et al. [98,99], the authors demonstrated that *Trem2* deficiency in mouse models of AD (*Trem2^−/−^* 5xFAD mice) resulted in a reduced clustering of microglia around Aβ plaques, leading to larger, more diffuse, and less dense plaques. Furthermore, they observed that these altered Aβ plaque characteristics were associated with increased toxicity to neural processes, as evidenced by a higher number of dystrophic neurites and hyperphosphorylated tau in *Trem2^−/−^* 5xFAD mice compared to 5xFAD mice. The increase in dystrophic neurites suggests greater damage to neuronal processes, including axons and dendrites, in the vicinity of Aβ plaques in the absence of TREM2. Similar findings were observed in the research by Yuan et al., in which *Trem2* deficiency interfered with the development of a neuroprotective microglia barrier, responsible for managing amyloid compaction and insulation [100]. These findings suggest that microglia expressing TREM2 play a crucial role in packing Aβ plaques and preventing damage to adjacent axons and dendrites, highlighting TREM2’s protective role in the early stages of Aβ deposition and limiting its spread and toxicity [98,99,100]. 

However, in other disease models, such as APPPS1-21, *Trem2* haplodeficiency has variable effects on Aβ accumulation as demonstrated by studies conducted by Jay et al. [101,102]. The authors showed that the complete deletion of *Trem2* in this model (*Trem2^−/−^* APPPS1-21) reduced Aβ burden at early stages but led to higher Aβ accumulation later on. This indicates that TREM2 deficiency can ameliorate amyloid pathology and be actually beneficial at early stages of the disease in APPPS1-21 mice but can be detrimental and exacerbate the disease as it progresses [103]. These contradictory findings between studies have raised questions within the field about whether *Trem2* deficiency has model-specific effects or different roles at various stages of pathology, given that the mice were examined at different points in the disease’s progression [101]. Despite these contradictions, there is a consensus that *Trem2* deficiency reduces microglia cell accumulation around plaques and attenuates inflammation-related gene expression [98,99,102]. Therefore, further research into the role of TREM2 in the context of AD is crucial, as it may reveal new therapeutic strategies.

#### 4.2.2. Therapeutic Targeting of TREM2 against Alzheimer’s Disease

Although a great interest in TREM2 as a therapeutic target for AD is emerging, it remains a challenging task, since TREM2 risk variants are found in less than 1% of the population, and it is still unknown whether potential TREM2 targeting could be effective in non-carrier AD patients [88]. Furthermore, the strategy for either activating or inhibiting TREM2 must be determined since different outcomes have been observed depending on the disease model used, the stage of the disease, and the pathological insult being studied. As previously mentioned, TREM2 activity ablation in the 5xFAD model has been shown to increase the burden and early onset of the pathology. Whereas, in other models, TREM2 ablation seems to have beneficial effects at earlier stages of the disease. Regardless of these concerns, TREM2 continues to attract interest due to its clear connection with the outcome and progression of AD. To date, the best strategy appears to involve stimulating TREM2 signaling in the early stages of the disease, before or at the beginning of amyloid deposition, and before tau pathology and neuronal loss. This approach leverages TREM2’s role in microglial stimulation and the transition from a homeostatic to an active state, which helps contain plaque formation, density, and size [88]. Modulation of TREM2 expression has been shown to decrease inflammation and phagocytosis promotion in vitro. Moreover, in vivo experiments showed that lentiviral approaches aiming to promote TREM2 overexpression in mice brains attenuated cognitive and neuropathologic alterations [104,105]. 

Immunotherapies using antibodies to stimulate TREM2 signaling are being developed. Schlepckow et al. developed the monoclonal antibody IgG1 (mAb) 4D9 that binds to and blocks the cleavage site of TREM2, stabilizing the protein and allowing its constant activation. The authors reported enhanced microglia capacity to engulf Aβ and neuronal debris and a reduction in amyloid deposition in a concentration-dependent manner [106]. This strategy is reported to be in initial clinical trials in combination with transferrin receptors that facilitate the crossing of the blood–brain barrier [107]. Another immunotherapy approach involves the activation of the TREM2 extracellular domain via the mAb IgG1 AL002, developed by the company Alector. Studies conducted by Wang et al. have shown a reduction in plaque size and better compaction in vivo [108]. In vitro studies using AL002 have demonstrated the activation of the TREM2 in microglia and an increase in their proliferative state. Currently, AL002 is in phase II of clinical trials, and it seems to be safe and well tolerated by participants, including healthy individuals and AD patients (NCT05744401). 

Another immunotherapy strategy, developed by Fassler, et al. is CGX101, an mAb IgG4 anti-TREM2 that aims to target the extracellular domain of TREM2 and shed TREM2 to constrain long-term chronic inflammation due to the activation of TREM2 by the agonizing mAb, as well as attenuate non-specific proinflammatory effects. Studies of CGX101 in vivo and in vitro have shown a reduction in amyloid burden in 5xFAD mice at early and later stages of the disease. Increased activated microglia coverage of the plaques, reduced cognitive decline, and reduced neuroinflammatory markers together with a reduction in dystrophic neurites were also observed [109]. 

### 4.3. The Complement System

The complement system is an ancient component of the immune system [110]. The discovery of the first complement proteins dates back to the late 19th century [111,112,113]. The modern terminology was concerted based on the order of reactions: C1, C4, C2, C3, C5, C6, C7, C8, and C9 in 1968 by the Complement Nomenclature Committee [114]. Beyond the classically described cascade, the integrated human complement system consists of more than 50 proteins, most of which are secreted by the liver and several of which can be differentially induced in a variety of other cell types and tissues, including the brain [115]. They are found either circulating in the blood or lymph or expressed on cell surfaces and, in some cases, on or in subcellular compartments [116,117]. The complement system is an integral part of the innate immune response and acts as a bridge between innate and acquired immunity [118]. The complement cascades can be initiated almost immediately upon pathogen entry and play a crucial role in controlling the early stages of infection. Three distinct activation pathways have been classically described; however, in reality, these pathways are closely interlinked [119]. Antibody-dependent activation, first described at the beginning of the 20th century, is now termed the classical pathway. The re-discovery in the 1950s of an antibody-independent, pathogen-triggered pathway for complement activation laid the foundations for the so-called alternative pathway. A third activation pathway, antibody-independent and triggered by pathogen-specific sugars, was described in the 1980s and is now known as the lectin pathway. 

However, the complement system is multifunctional and far beyond infection defense. For example in the CNS, it was demonstrated to be fundamental in several physiological processes [120], including shaping neuronal networks during early development through highly controlled synaptic pruning [121], assisting in appropriate neuronal migration, polarization, and proliferation [122,123], and influencing neuronal excitability through the modulation of neuronal intracellular calcium transients [124]. In the adult brain, the complement system has a significant influence, as the forgetting of remote memories relies on a complement-dependent synaptic elimination mechanism mounted by microglia [125]. The complement system as a fundamental part of the innate immunity is a double-edged sword that usually protects the host but, under chronic or overactivation conditions, can cause tissue damage such as in the case of neurodegenerative conditions [126]. 

#### 4.3.1. Complement System in Alzheimer’s Disease

The complement system has been increasingly implicated in the pathogenesis of AD. While the complement rarely constitutes the sole driver of disease, it acts as an initiator, contributor, and/or exacerbator in numerous disorders [127]. Its contribution to pathology and dysfunction is complicated by region- and time-specific activation as well as differences in animal models used to investigate these processes [128]. A bioinformatic analysis by Xi-Chen Zhu et al. confirmed that more than 20 genes were closely related to differentially expressed complement system components in AD after intersecting the disease-related complement system module genes and differentially expressed genes [129]. In many CNS diseases, complement proteins are significantly upregulated before signs of neuronal loss occur, which suggests that the reactivation of mechanisms similar to complement-mediated synaptic elimination may drive disease progression [121].

Synapse loss is considered the strongest neurobiological correlate of cognitive impairments in AD [130,131]. According to current findings, C1q and C3 play an important role in this process, probably through direct binding to their receptors at synapses or transport via extracellular vesicles from microglia and astrocytes to synapses [132,133,134]. C3, the central component of the complement system, has also been reported to impact synaptic density impairment through the neuronal complement receptor C3aR in the astrocyte–neuron co-culture system [135]. An essential role for C3 in promoting microglia-dependent synapse elimination during aging has been confirmed in aged *c3^−/−^* mice, which display attenuated synaptic elimination in the hippocampus resulting in improved cognitive performance [136]. In addition, complement proteins found on synapses may also be synthesized within neurons or synapses [137]. Whether complement proteins deposited at synapses in AD come from the neurons themselves or from glial cells remains to be clarified. A recent study showed that the optogenetic activation of microglia promoted the clearance of Aβ in the brain parenchyma, but also increased synaptic elimination. The researchers further demonstrated that this synaptic elimination was dependent on C1q [138].

The first piece of evidence linking the complement to AD came from studies, in which plaques and periplaque areas stained positive for complement proteins and activation products [126,139]. A study by Spurrier et al. showed that inhibiting oligomeric Aβ-mediated neuronal damage prevented C1q deposition at the neuronal synapse, resulting in attenuated pathology and cognitive decline in mouse models of AD [140]. C1q, the initiating protein of the classical complement cascade, is increased and associated with synapses before plaque deposition [141]. It regulates phagocytosis and synapse clearance by microglia, contributing to synapse loss in AD patients [142]. Notably, early and locally produced C1q in response to injury, without the coordinated expression of other complement components, may have a protective role and limit the progression of disease [128,143,144,145]. Downstream complement activation leads to neurotoxicity [128]. Moreover, the classical complement system can also be activated by p-tau found in NFTs [146]. The activation of the classical complement pathway leads to the deposition of C3b, formation of the C3 and C5 convertases, and production of C3a and C5a anaphylatoxins. The role of C3aR in AD is complex, as reported data have suggested both protective and detrimental effects. For example, the genetic ablation of *C3ar1* ameliorates disease in tauopathy models [147], but it also enhances neurogenesis in the adult brain [148]. This dual role for the complement in AD has wide therapeutic implications. 

#### 4.3.2. Complement-Targeting Therapy in AD

The increasing number of complement-targeting drugs that have been approved or are in advanced clinical trials is encouraging and starting to benefit a growing number of patients. However, none of the approved complement-targeting drugs have been yet tested in AD patients [110]. Inhibitors of complement activation have been studied in animal models. Treatment with a C5aR antagonist decreases pathology and enhances behavioral performance in murine models of AD. C5a and fibrillar Aβ together injure neurons more than either protein alone. The additive damage to neurons can be blocked by PMX53, a C5aR1-specific antagonist [149]. Research by Fonseca et al. showed that the oral delivery of a cyclic hexapeptide (PMX205), another C5aR1 antagonist, for 2–3 months resulted in a substantial reduction in pathological markers such as fibrillar amyloid deposits (49–62%) and activated glia (42–68%) in two mouse models of AD [150]. An improvement in the passive avoidance behavior task in Tg2576 mice was correlated with reduced pathology. In 3xTg-AD mice, PMX205 also significantly reduced p-tau (69%) [150]. Similar results were found recently by Gomez-Arboledas et at. in the Tg2576 mouse model [151]. Additionally, the intermittent oral administration of an additional C5a receptor antagonist (EP67) has been shown to enhance microglia phagocytosis and subsequently reduce Aβ and astrocytosis and rescue memory deficits and synaptic markers in 5xFAD mice [152]. Taking together these results, it is safe to propose that the use of already approved C5aR1 antagonists such as Avacopan (ChemoCentryx) must be tested in preclinical and clinical studies of AD patients. 

Another complement-based target in AD is the natural inhibitor component 8 gamma (C8G), whose expression has been found to be upregulated mainly in astrocytes of postmortem tissue of AD cases. The intrathecal delivery of recombinant C8G in 5xFAD mice ameliorated neuroinflammation and behavioral deficits, whereas C8G knockdown exacerbated AD pathology [153]. Targeting the complement system seems to be a feasible and promising strategy for future clinical trials in the preclinical phases of AD, especially considering the vast repertory of compounds already tested or being tested in other immune-related conditions. 

### 4.4. Inflammasomes

The most well-understood PRRs involved in neurodegeneration are the TLRs, CDSs, NLRs, and ALRs, which can form inflammasome complexes. Inflammasomes are multiprotein complexes that play a central role in the innate immune response by regulating the production of pro-inflammatory cytokines and initiating inflammatory responses. They act as a molecular platform within cells, particularly immune cells, in response to various danger signals, including microbial pathogens, toxins, and DAMPs [154]. Inflammasome complexes typically consist of three key components: a cytosolic pattern-recognition receptor or sensor molecule, the enzyme caspase 1 or effector molecule, and an adapter protein that enables contact between the two, known as an apoptosis-associated speck-like protein with a CARD (ASC). There are various types of inflammasomes, which differ in the composition of their sensor molecule and in the requirement or lack thereof for an adaptor molecule [154,155]. The NLRs contain a carboxy-terminal leucine-rich repeat (LRR) domain, a conserved central NACHT domain (essential for protein oligomerization and required to form multiprotein inflammasome complexes), and a variable amino-terminal domain that defines several NLR subfamilies. NLRs carrying an N-terminal pyrin domain (PYD) make up the NLRP subfamily, while NLRs with an N-terminal caspase activation and recruitment domain (CARD) constitute the NLRC subfamily. NLRPs rely on the adaptor protein ASC, which is also referred to as PYCARD because it contains two domains, a pyrin domain (PYD) and a CARD, acting as a molecular platform that connects the PYD of the NLRP sensor with the CARD of pro-caspase 1. Conversely, NLRCs can directly interact with pro-caspase 1 through their respective CARD domains, rendering ASC unnecessary for inflammasome activation. The NLRP1 downstream pathway stands as the sole exception to the conventional activation of NLRPs, as it, along with the NLRCs, does not require the adapter to interact with pro-caspase 1 [154,156,157].

#### 4.4.1. Inflammasome Activation

Inflammasome activation typically follows a two-step process. The initial step, known as “priming”, involves the activation of cell surface receptors like TLRs upon exposure to danger signals. This triggers the activation of the myeloid differentiation primary response protein (MyD88)-nuclear factor-κB (NF-κB) pathway, leading to the transcription and synthesis of key inflammasome components and precursor forms of pro-inflammatory cytokines. The second step encompasses the assembly and activation of the inflammasome complex itself. Upon the detection of danger signals, the sensor molecule undergoes conformational changes and recruits ASC, facilitating inflammasome assembly. Once assembled, the inflammasome activates pro-caspase-1, initiating its cleavage. Activated caspase-1 processes pro-inflammatory cytokines such as pro-IL-1β and pro-IL-18 into their active forms, IL-1β and IL-18, respectively. Simultaneously, caspase-1 cleaves the protein Gasdermin-D, leading to its activation and insertion into the cell membrane, forming pores. These pores facilitate the release of IL-1β, IL-18, and other intracellular contents, triggering inflammation and attracting immune cells to the site of infection or injury. Sustained pore formation results in cellular swelling and ultimately pyroptosis, a highly inflammatory form of cell death [154,158,159]. In addition to the inflammasome complex formation, inflammasome activation can also result in ASC “specks” formation as the adapter protein ASC can get polymerized and aggregate via homotypic interactions between its PYD domains into large fibrils. These ASC fibrils cross-link via interactions between its CARD domains, forming supramolecular aggregates called “specks” that serve as platforms for the further recruitment of caspase-1, enhancing the inflammatory response [160,161,162]. 

#### 4.4.2. Relationship between Inflammasomes and Alzheimer’s Disease Pathogenesis and Progression

The concept of inflammasomes playing a role in propagating inflammation and exacerbating the course of diseases was first proposed by Jürg Tschopp and colleagues in the early 2000s [163,164] when they elucidated the structure and function of the first inflammasome NLRP1, demonstrating their involvement in inflammatory processes associated with various diseases. After that, many inflammasome complexes have been examined in the disease context, including neurodegeneration and AD, such as AIM2. However, in the brain, NLRP3 is the most studied inflammasome complex to date [165]. Several studies [166,167,168,169] have demonstrated that Aβ and tau can activate microglial NLRP3 inflammasomes upon phagocytosis by TLRs and other scavenger receptors. Once Aβ is internalized, it can disrupt lysosomes, being released into the cytosol. This intracellular presence of Aβ triggers the assembly of the NLRP3 inflammasomes, leading to the production of cleaved-caspase-1 and IL-1β and the release of proinflammatory cytokines. This sustained neuroinflammation in the brain can exacerbate neuronal damage and accelerate the cognitive decline observed in AD patients. Moreover, chronic inflammation and the persistent release of inflammatory cytokines can enhance both Aβ and tau pathologies by disrupting the normal processing of amyloid precursor protein (APP), promoting the formation and aggregation of toxic Aβ oligomers. Additionally, it fosters the abnormal phosphorylation and aggregation of tau protein, ultimately resulting in the formation of NFTs. [167,168,170]. Furthermore, recent studies have shown that the NLRP3 inflammasome is not only activated by fibrous Aβ aggregates but also by lower-molecular-weight Aβ oligomers and fibrils, suggesting that the innate immune response of the CNS triggered by Aβ may occur before the onset of Aβ plaques [171,172]. These studies support the hypothesis that inflammation is not merely a consequence of AD pathology, but rather a contributing factor that actively promotes the progression of the disease.

In 2014, Franklin et al. [173] introduced a groundbreaking concept in the field, suggesting that following inflammasome activation, ASC specks are released into the extracellular matrix through either pyroptotic or non-pyroptotic pathways. These specks remain functionally active, facilitating the recruitment of additional phagocytic cells to the site of inflammation. Once in the extracellular matrix, ASC specks can be taken up by phagocytic cells, where they may undergo degradation or function as exogenous danger signals, further amplifying inflammasome activation and propagating inflammation in a prionoid-like manner, similar to fibrillar protein aggregates like Aβ, tau, and α-synuclein [173]. Additionally, studies have indicated that ASC specks possess an inherent ability to co-aggregate cytosolic proteins on their surface through non-specific hydrophobic interactions, suggesting a potential role for ASC as a scaffold for other aggregation-prone proteins [162,174]. In 2017, Venegas et al. [170] showed that extracellular ASC specks directly promote Aβ aggregation both in vitro and in vivo. Cultured mouse microglia release ASC specks upon exposure to Aβ. Moreover, purified ASC specks incubated with Aβ accelerated the aggregation speed in a concentration-dependent manner. These in vitro findings were confirmed with in vivo experiments. An immunohistochemical analysis of postmortem human brain tissue and APP/PS1 mice revealed the presence of ASC aggregates within the core of extracellular Aβ plaques. Notably, APP/PS1 crossed with *Asc^−/−^* mice exhibited reduced Aβ plaque deposition and the attenuation of memory deficits. Furthermore, the injection of APP/PS1 brain lysates into the hippocampus of APP/PS1 mice led to an increase in the size and number of Aβ plaques compared to the non-injected contralateral side; this seeding effect was reduced in *Asc^−/−^* mice or when a neutralizing ASC antibody was co-injected with the brain lysate. Collectively, these findings underscore the role of ASC aggregates as scaffolds for the cross-seeding of Aβ, exacerbating amyloid pathology [170].

#### 4.4.3. Therapeutic Targeting of Inflammasomes in AD

Targeting inflammasome components represents a promising therapeutic strategy against AD by addressing the neuroinflammatory processes that exacerbate disease pathology. Studies [20,167,168,170] have shown that the inhibition of NLRP3, ASC, Caspase-1, or IL-1β can reduce Aβ deposition, tau pathology, and neuroinflammation, suggesting that targeting these components can mitigate the neurodegenerative processes underlying AD. Heneka et al. demonstrated that *Nlrp3^−/−^* and *Caspase-1^−/−^* APP/PS1 mice were protected from neurobehavioral disturbances compared to APP/PS1 mice with an intact NLRP3 inflammasome cascade. Additionally, hippocampal synaptic plasticity was preserved, and the Aβ plaque load was reduced in both the hippocampus and cortex, with the enhanced microglial phagocytosis of Aβ plaques [167]. Furthermore, Ising et al. showed reduced levels of p-tau in *Nlrp3^−/−^* crossed with the Tau22 mouse model [168,170]. Despite the extensive evidence, these results have been recently challenged by Srinivasan et al., as conditional or global *Nlrp3* and *Caspase-1* knockout failed to rescue AD pathology in *App^NL-G-F^* and APP/PS1 mice [175]. Nevertheless, based on these findings, several inhibitors, antibodies, and therapies are being developed at pre-clinical and clinical trial stages, showing promising results and offering a hopeful outlook for the treatment of AD. 

##### NLRP3 Inhibitors

Small-molecule inhibitors targeting the NLRP3 inflammasome, such as MCC950 (also known as CRID3) [176,177], CY-09 [178], and OLT1177 (dapansutrile) [179], have shown efficacy in preclinical models of AD by attenuating inflammasome activation, reducing neuroinflammation, and ameliorating cognitive deficits [165,171]. These inhibitors block NLRP3 assembly or activation, thereby suppressing the release of the pro-inflammatory cytokines IL-1β and IL-18. MCC950, developed by the pharmaceutical company Pfizer, and CY-09, developed by a research team led by Dr. Hongbo Luo, both inhibit NLRP3 by targeting its NACHT domain. This domain is crucial for the ATP/GTPase activity necessary for the activation and oligomerization of NLRP3 [176,178,180]. Preclinical studies of MCC950 conducted in APP/PS1 mice and Long-Evans rats infused with Aβ oligomers have demonstrated an improvement in cognitive function compared to vehicle-treated counterparts. Additionally, MCC950 treatment resulted in reduced Aβ plaque accumulation, a decreased activation of microglial cells, and lower levels of pro-inflammatory cytokines [177,181]. CY-09, although primarily studied in other inflammatory diseases, has shown promise in reducing NLRP3-related inflammation [178,182]. OLT1177 (dapansutrile), developed by the pharmaceutical company Olatec Therapeutics, binds specifically and strongly to NLRP3, interfering with the oligomerization process [179]. In recent studies performed in APP/PS1 mice, OLT1177 rescued spatial learning and memory, reduced microglia activation as well as cortical plaque burden, and normalized plasma AD metabolic markers [183]. Moreover, OLT1177, although focused on other inflammatory diseases, is currently in several clinical trials for acute gouty arthritis [165,171]. 

Other NLRP3 inhibitors tested in pre-clinical stages for AD include tranilast [184], JC124 [185,186], oridonin (Ori) [187], and BAY 11-7082 [188]. Tranilast, initially developed as an anti-allergic drug by Kissei Pharmaceutical Co., was repurposed as a direct NLRP3 inhibitor that can inhibit its oligomerization and NLRP3–NLRP3 interactions. Studies have reported that Tranilast can improve cognitive behavioral parameters and significantly increase memory-related proteins in Aβ-induced cognitive deficit model mice [171,189]. JC124, developed by MedKoo Biosciences, has demonstrated promising results in studies conducted on APP/PS1 mice and CRND8 APP transgenic mice. It improved cognitive function and reduced Aβ deposition, accompanied by a decreased β-cleavage of APP, reduced microglial activation, and enhanced astrocytosis. Furthermore, JC124 treatment decreased oxidative stress, indicating a possible neuroprotective effect [185,186]. Oridonin (Ori), the main bioactive component of the natural anti-inflammatory Chinese medicinal herb Rabdosia rubescens, has been identified as a specific covalent inhibitor of the NLRP3 inflammasome [171,187]. Ori has shown efficacy in reducing pro-inflammatory cytokine levels and inhibiting the NF-κB signaling pathway in cultured neuronal cells treated with Aβ to induce neuroinflammation. In mice treated with Aβ to induce Aβ-like pathology, Ori inhibited microglial activation, reduced inflammatory cytokine release, prevented synaptic loss, and improved cognitive impairment [190,191]. 

Other NLRP3 inhibitors in clinical trials at different stages, although not tested in AD, that could represent a possible therapy against neuroinflammation and neurodegenerative disorders include DFV890 (IFM-2427), selnoflast, inzomelid (IZD174), NT-0796, RRX-001, VTX2735, and ZYIL1 [165].

##### ASC Speck Disruption

Studies suggest that targeting and disrupting the assembly and polymerization of ASC specks can reduce inflammation and treat inflammatory diseases [192], like gout, arthritis [193,194], and multiple sclerosis [195]. While this strategy has not been thoroughly tested in AD so far, drug-targeting ASC speck formation could potentially mitigate inflammasome-mediated pathology in AD, as observed in AD mice models with ASC deficiency (*Asc^−/−^*) [168,170]. Various approaches, including small-molecule inhibitors and antibodies, have shown promise in this regard. For instance, small-molecule inhibitors of ASC have emerged as an attractive option to treat inflammatory diseases. MM01 [192], a novel small-molecule inhibitor of ASC oligomerization, has demonstrated a highly specific inhibition of ASC-mediated pro-caspase-1 activation, accompanied by ASC speck disruption, and a reduced secretion of IL-1β and IL-18 in in vitro models. In a mouse model of inflammasome-induced peritonitis, MM01 significantly reduced inflammation, suggesting the potential of MM01 to treat multifactorial diseases involving the dysregulation of inflammasomes [192]. Similarly, antibodies against ASC have been explored as potential treatments for various inflammatory diseases. IC100, a novel humanized monoclonal IgG4 antibody targeting ASC, developed by ZyVersa Therapeutics, has been shown to improve functional outcomes in animal models of multiple sclerosis (MS) by reducing disease severity and suppressing immune cell infiltration in the CNS [195]. Taken together, these studies confirm that disrupting ASC specks represents a promising therapeutic strategy for treating various inflammatory diseases and has great potential for clinical applications.

Other strategies to disrupt ASC speck formation and inhibit NLRP3 inflammasome activation that have been tested in AD models and patients involve modulating their metabolism through ketogenic diets, caloric restriction, or fasting [196]. Studies have shown that the long-term administration of ketogenic formulas can improve cognitive function in AD patients [196,197]. A specific ketone body produced during such metabolic changes, β-hydroxybutyrate (BHB), is of special interest since it has been shown to be able to inhibit NLRP3 inflammasome activation by preventing K+ efflux and reducing ASC oligomerization and speck formation [196]. In pre-clinical studies conducted in the 5xFAD mouse model of AD, reduced plaque and ASC speck formation as well as reduced caspase-1 activation was observed after the exogenous administration of BHB [198]. These findings suggest that BHB could alleviate AD pathology. In addition, the capability of BHB to cross the BBB increases its attractiveness and therapeutic potential as a treatment strategy against AD [171]. 

##### Caspase-1 Inhibitors

Caspase-1 inhibitors hold promise as potential therapeutic targets for AD treatment, either alone or in combination with other therapeutic approaches. They exert anti-inflammatory effects by reducing the production and release of pro-inflammatory cytokines, potentially preserving neuronal function and slowing disease progression. Studies have identified potential caspase-1 inhibitors that show potential in preclinical AD studies, including VX-765 [199,200,201] and Ac-YVAD-cmk [202,203,204]. Caspase-1 inhibitors work by binding to the active site of caspase-1, preventing the enzyme from cleaving and activating pro-inflammatory cytokines [205]. VX-765, developed by Vertex Pharmaceuticals, is a non-toxic inhibitor that can permeate the blood–brain barrier (BBB). Studies conducted in AD mice models have shown that VX-765 can prevent progressive Aβ deposition and reverse brain inflammation, synaptic loss, and memory impairment. The preventive usage of VX-765 before the onset of AD symptoms in the AD J20 mice model has shown a delay in cognitive impairment [199,201]. Moreover, VX-765 has shown promise in other central nervous system (CNS) disorders, such as models of multiple sclerosis [200] and epilepsy, with ongoing phase II clinical trials [171]. Ac-YVAD-cmk is a highly selective, cell-permeable, potent, and irreversible inhibitor against caspase-1. It has been shown to be effective in reducing inflammation in the CNS, inhibiting cell pyroptosis, improving neurological function, and providing a neuroprotective effect in various animal models, including for intracerebral hemorrhages [202,203]. In AD, Ac-YVAD-cmk treatment has been shown to improve spatial learning and memory impairment in APP/PS1 mice and reduce Aβ plaque deposition [204].

### 4.5. Cytosolic DNA Sensors

The sensing of DNA as a dangerous signal has been known for many decades, and it plays an important role in the fast activation of an immune response upon infection. Nucleic acids from microbes, either viruses or bacteria, induce an antiviral response characterized by the release of type I interferons (IFN I) and the induction of interferon-stimulated genes (ISGs) and other pro-inflammatory mediators, restricting the propagation and spread of the infection [206,207,208]. In recent years, it has been shown that the sensing of endogenous DNA can serve as a surveillance mechanism of cellular stress signals, including failure with autophagy, mitochondrial stress with mitochondrial-DNA (mtDNA) leakage, or genotoxic DNA damage with genomic DNA (gDNA) cytosolic exposure [209,210,211,212]. This aspect holds particular importance in cells that present higher DNA damage response activation, like epithelial cells being exposed to UV light, cancer, and senescent cells, or that are more vulnerable to mitochondrial stress, like nerve and heart cells.

Cytosolic DNA sensors include various protein complexes that have different structures, DNA binding modes, molecular activation, cell type distributions, changing expression levels, and locations within the cell, characteristics that are related to their biological effects on health and disease. They all share the common function of detecting foreign DNA and self-DNA and triggering a type I interferon response [213,214]. In mammals, the cyclic GMP–AMP synthase (cGAS) appears to be the most important innate immune DNA sensor. Upon its activation by DNA binding, cGAS undergoes conformational changes allowing for the enzymatic production of 2′ 3′ cyclic GMP–AMP (cGAMP). This molecule acts as a secondary messenger and potent activator of stimulator of interferon genes protein (STING). The identification of cGAS and its enzymatic function in producing cyclic dinucleotide represented the missing link between DNA detection and STING activation, which was already described as important for the antiviral response against pathogenic DNA [215,216,217,218,219]. Studies over the past years have shed light on the functioning of this enzyme. In summary, the C-terminal region of human cGAS (h-cGAS) represents the catalytic section and presents positively charged DNA binding sites. These consist of a primary site and two additional sites that interact with DNA. When DNA binds to the primary site, it triggers conformational changes in the protein that rearrange the catalytic pocket, facilitating an optimal interaction with ATP and GTP substrates. The binding of DNA to the adjacent B-site is essential for forming the core 2:2 cGAS-DNA complex, which is the minimal active enzymatic unit. On extended dsDNA molecules, cGAS dimers organize into ladder-like networks and phase-separated organelles. This activation mechanism provides a vital safeguard for cells: cGAS signaling is only initiated when longer dsDNA stretches are present in the cytoplasm, ensuring a sufficient signaling threshold is reached. Normally, the DNAs of human self-cells are not exposed enough to trigger cGAS, and this is due to a tight regulation of this DNA sensor’s activity by enzymatic degradation or binding to inhibitors, avoiding inducing autoimmunity [220,221].

Once cGAMP is produced by cGAS it quickly diffuses throughout the cell and binds to STING, a transmembrane protein located on the endoplasmic reticulum (ER). STING consists of a short cytosolic N-terminal segment, a four-span transmembrane domain, a connector region, and a cytosolic ligand-binding domain (LBD) with an appended C-terminal tail (CTT). STING is synthesized, modified, and resides as a homodimer in the ER. cGAMP binding causes conformational changes that allow for the exit of STING from the ER, and through ER–Golgi intermediate compartment (ERGIC) translocation to the Golgi [208,222,223,224,225]. These changes permit the binding of tank-binding kinase 1 (TBK1) and its activation, with the phosphorylation of TBK1 and STING. Phosphorylated STING binds to a positively charged region of the transcription factor interferon regulatory factor 3 (IRF3), allowing for its phosphorylation by TBK1. Phosphorylated IRF3 forms dimers and translocates into the nucleus to activate IFN genes and ISGs. Besides activating IFNs, STING has also been reported to activate NF-κB-mediated transcription, inducing the expression of proinflammatory cytokines and chemokines, such as TNF-α, IL-1β, IL-6, CXCL10, CCL5, and others [208,226,227,228].

Before the discovery of cGAS, other DNA sensors were described. Among them, interferon-gamma-induced protein 16 (IFI16, also known as P204 in mice) and absent in melanoma 2 protein (AIM2) both belong to the pyrin and hematopoietic interferon-inducible nuclear (PYHIN) family [214,229,230,231]. Similar to cGAS, AIM2 and IFI16 bind with DNA in a sequence-independent and a length-dependent manner. Instead of producing a secondary messenger to induce STING activation, these sensors can bind through PYD-PYD interaction with ASC to form inflammasomes and the induction of pro-inflammatory response, as previously described. IFI16 bound to DNA has also been shown to directly bind with STING, trigger the TBK1-IRF3 pathway, and cooperate with cGAS to sense DNA and amplify its response [232,233]. Other researchers proposed that IFI16 plays a role in inhibiting the cGAS-mediated immune effect by competing for DNA [234]. IFI16 also acts as a dual regulator in p53-mediated cell checkpoint control and has been shown to play an important role in cell cycle arrest, senescence, and apoptosis [229]. Other less-explored cytoplasmic DNA sensors are DNA-dependent activators of IFN-regulatory factors (DAI), also known as DNA-binding protein 1 (ZBP1) and DEAD-box helicase 41 (DDX41). ZBP1 mainly binds with Z-form DNA, and upon activation, both receptor-interacting protein kinase 1(RIPK1) and RIPK3 can bind via homotypic interactions with receptor-interacting protein homotypic interaction motifs (RHIMs). Lastly, the C-terminus also contributes to ZBP1-mediated IFN-I production by recruiting the TBK1-IRF3 complex [235]. In contrast, DDX41 binds to a single strand of DNA and mainly triggers STING-TBK1-IRF3-IFNβ signaling through direct STING binding [236]. 

#### 4.5.1. GAS-STING Pathway in AD

Growing evidence has indicated that dysregulation in DNA sensing, particularly from the cGAS-STING axis, plays an important role in driving inflammatory responses in a number of neurodegenerative diseases, including amyotrophic lateral sclerosis (ALS), Parkinson’s disease (PD), Huntington’s disease (HT), TBI, and FTD, and more recently, AD [237,238,239,240,241,242,243,244,245]. Pathological features of neurodegenerative diseases include inflammation, protein aggregation, defective proteostasis, defects in DNA and RNA homeostasis, altered energy metabolism, synaptic and neuronal network dysfunction, cytoskeletal abnormalities, and neuronal cell death, all of which can influence the release of self-DNA into the cytoplasm and lead to the activation of cGAS under sterile conditions. For example, the loss of genomic integrity during DNA replication or damage can lead to the formation of micronuclei, which can rupture and release chromosomal DNA into the cytosol, triggering cGAS activation. Similarly, cytoplasmic chromatin fragments (CCFs) can accumulate through the autophagic removal of nuclear DNA or DNA leakage from the nucleus during cellular senescence and aging. Additionally, mitochondrial stress or damage can release mitochondrial DNA (mtDNA) into the cytosol. cGAS can also detect DNA/RNA hybrids, which can originate from the retrotranscription of retroviruses or endogenous retroviral elements, among other sources. In the CNS, cGAS and STING are mainly found in microglial cells, but recent findings have shown expression in other cell types, such as astrocytes, oligodendrocytes, and neurons under specific conditions, such as chronic inflammation and aging [212,246].

In order to identify a relationship between the type I IFN signature observed in AD patients and cGAS signaling, researchers have studied the role of this immune pathway in multiple models of AD [247,248,249,250]. Elegant results showing microglial cGAS-STING activation were obtained using the 5xFAD model, in which nucleic acid-containing Aβ plaques induced an interferon response. Blocking IFNAR with an inhibitory antibody significantly reduced microglial activation, highlighting the involvement of nucleic acid-sensing innate immune pathways in AD pathogenesis [247]. Similar results were obtained by Xie et al., who observed that cGAS binds cytosolic dsDNA and becomes activated in human AD brains. Furthermore, they found phosphorylated STING and IRF3 colocalized with the activated microglia marker CD68 around Aβ plaques in the dentate gyrus of 5xFAD mice, suggesting cGAS-STING pathway activation. Deleting cGAS in these mice alleviated cognitive impairment, reduced Aβ pathology, decreased ISG expression in the hippocampus, and inhibited neurotoxic reactive astrocyte formation. Additionally, Aβ-induced neurotoxicity was partially mitigated with *Cgas^−/−^* astrocyte-conditioned media, indicating that cGAS-STING may regulate the neuroinflammatory response via a microglia–astrocyte–neuron signaling pathway [214]. Studying the APP/PS1 amyloid mouse model, Hou et al. found that the induced inflammation in their brains was correlated with reduced levels of NAD+ and that treatment with an NAD+ precursor increased brain NAD+ levels, reduced the expression of proinflammatory cytokines, and decreased the activation of microglia and astrocytes, with reduced NLRP3 inflammasome expression, DNA damage, apoptosis, and cellular senescence in the AD mouse brains, dependent on the cGAS-STING [251].

Polyglutamine binding protein 1 (PQBP1) has also been related to inflammation towards tau protein. PQBP1, a sensor of the cGAS-dependent innate response to HIV-1, binds to reverse-transcribed HIV-1 DNA and interacts with cGAS. Extrinsic tau proteins were found to bind to PQBP1 and activate microglia in a cGAS-STING-dependent manner and induce the nuclear translocation of NFκB in vitro. In vivo, the microglia-specific deletion of *Pqbp1* prevented PQBP1 colocalization with tau, the nuclear translocation of NFκB, cGAS recruitment, reduced inflammatory gene expression, and rescued cognitive deficits in an acute tau protein injection model [239]. A recent study found that cGAS-STING is activated in the P301S tauopathy mouse model and human AD brains with high tau pathology levels. They observed that pathogenic tau activates cGAS in microglia, partly by causing mtDNA to leak into the cytosol, with the induction of IFN-inducible cytokines like CXCL10 and CCL5 and the upregulation of ISGs such as Stat1, Trim30a, and Ddx60. Deleting *Cgas* in the P301S mice reduced tauopathy-induced microglial IFN-I, protected against synapse loss, synaptic plasticity deficits, and cognitive impairment, though it did not affect tau pathology levels. Single-nucleus RNA sequencing of excitatory and inhibitory neuronal populations in P301S *Cgas^−/−^* hippocampi revealed the robust restoration of MEF2C transcriptional targets, a key factor in cognitive resilience against AD pathology [240]. The promising results obtained from pre-clinical models of AD demonstrate that cGAS and STING are unconventional but potentially druggable therapeutic targets covering different inflammatory mechanisms of action compared with current approaches to treat AD.

#### 4.5.2. Therapeutic Targeting of the cGAS-STING-IFN Axis

The cGAS and STING inhibitors that have been reported to date and are advancing in clinical development were mainly investigated in the context of cancer [252]. The pharmacological inhibition of cGAS by the brain penetrant molecule TDI-8246 in mice with tauopathy restored synaptic integrity, plasticity, and memory. This was accompanied by an enhanced neuronal MEF2C transcriptional network, suggesting the cGAS-IFN-MEF2C axis bolsters resilience against AD-related pathological insults [240]. The STING inhibitor H-151 was also reported to suppress the activation of the cGAS-STING pathway and ameliorate AD pathogenesis in 5×FAD mice [242]. These results coincide with the reported effects of this inhibitor in aging mice, in which the blockade of STING suppressed the inflammatory phenotypes of senescent human cells and tissues, including mice brains, and led to an improvement in tissue function [253]. This points out the important relationship between aging, the cGAS-STING pathway, and AD development.

## 5. Conclusions

Neuroinflammation is not only a mere contributor but can also be a driving force behind AD pathophysiology and progression. Several studies testing antibodies, inhibitors, agonists, and diets targeting neuroinflammation have brought hope for improved therapy in AD (Table 1). However, the complexity of immune interactions poses an important obstacle to address in drug research. Acute neuroinflammation is necessary for maintaining brain homeostasis while the sustained overactivation of the innate immune system can be a priming factor for disease development and exacerbate its progression. A great example of this are microglia, which are crucial in brain development, in maintaining homeostasis, and in the response to DAMPs/PAMPs, including the clearance of Aβ. However, their chronic activation leads to impaired Aβ removal, synapse engulfment, and neuronal damage, resulting in neurodegeneration. Thus, it is necessary to find the right balance of specific immune system components to avoid detrimental side effects. Moreover, it is important to consider the best time window and the administration routes as well as interactions with genetic and environmental factors to increase the probability of success in clinical studies. However, the intricacy of the immune system has also advantages as it presents multiple promising targets for therapeutic modification: from specific immune cell modulators, through pro-inflammatory cytokines to multiprotein complexes. Therefore, the further understanding of the innate immune system within the context of neurodegeneration should be the main focus as it provides new directions for medical intervention in AD.

## Figures and Tables

**Table 1:** Innate immune targets to tackle AD.

Target	Endogenous Ligands	Physiological Effects	Potential Compounds for AD	Ref.
CSF1R-1	CSF-1, IL-34	Survival and proliferation of microglia	Inhibitors: PLX3397 (PEXIDARTINIb); PLX5622, BLZ945, Ki20227, JNJ-527, ARRY-382, and GW2580. Agonists: CSF-1	[39,40,41,42,43,44,45,50]
IL-10RB	IL-10	Decreases proinflammatory activity of microglia	Agonists: IL-10, AAV–IL-10	[53,58]
CD200R	CD200	Inhibitory effect on microglia activity	CD200, AAV-CD200	[63]
CX_3_CR1	Fractalkine (CX_3_Cl1), sCX_3_Cl1	Synaptic pruning during development, control of neuronal network stability, synaptic plasticity, anti-inflammatory	Agonists: AAV-sCX_3_Cl1, Tet34 Inhibitors: AZD8797, MAB E6011	[80,82,86]
TREM2	PS, PE, LDL, S1P, ApoE, or Aβ	Regulation of microglia survival, activation, and phagocytosis.	Antagonist: 4D9, AL002, CGX101	[106,107,108,109]
Complement system	Antibodies	Synaptic pruning, neuronal migration, polarization and proliferation, synaptic elimination, forgetting of remote memories	Inhibitors: PMX205, EP67, Avacopan (C5aR1 antagonists), recombinant C8G	[150,151,152,153]
NLRP3	Aβ, MSU, cholesterol crystals, ATP, cation currents	Neuroinflammatory responses	Inhibitors: MCC950, OLT1177, CY-09, Tranilast, JC124, Oridonin, BAY 11-7082, DFV890 (IFM-2427), Selnoflast, Inzomelid (IZD174), NT-0796, RRX-001, VTX2735, and ZYIL1	[165,176,177,178,179,183,187,188]
ASC	Inflammasome activators	Unique adaptor protein for inflammasomes	MM01, IC100	[192,195]
Caspase-1	ASC and other inflammasome activators	Cleavage of IL-1β, IL-18, and Gasdemin D	VX-765, Ac-YVAD-cmk	[199,200]
cGAS- STING	Cytoplasmic DNA, p-tau	Induction of type I IFN responses	cGAS inhibitor: TFI-8246 STING inhibitor: H-151	[240,253]

Abbreviations: AAV: Adenovirus-associated virus vectors; ApoE: Apolipoprotein E 4; ASC: Apoptosis-associated speck-like protein containing a CARD; Aβ: Amyloid β; cGAS: Cyclic GMP-AMP synthase; CR200R: Cluster of Differentiation 200 (CD200) receptor; CSF1R: colony stimulating factor 1 (CSF1) receptor; CX3Cl1: C-X3-C motif chemokine ligand 1; CX3CR1: C-X3-C motif chemokine receptor 1; IFN: Type I interferon; IL-10 RB: Interleukin 10 receptor (IL-10) subunit beta; IL-34: Interleukin 34; LDL: Low-density lipoprotein; MSU: Monosodium urate; NLRP3: Nucleotide-binding oligomerization domain, Leucine rich repeat and pyrin domain containing 3; PE:phosphatidylethanolamine; PS: Phosphatidylserine; S1P: Sphingosine-1-phosphate; sCX3Cl1: Soluble C-X3-C motif chemokine ligand 1; STING: Stimulator of interferon genes protein; TREM2: Triggering receptors expressed on myeloid cells 2.

## Data Availability

Not applicable.

## Abbreviations

AAV	Adenovirus-associated virus vectors
AD	Alzheimer’s disease
ADAM10	Disintegrin and metalloproteinase domain-containing protein 10
AIM2	Interferon-inducible protein absent in melanoma 2
ALR	Absent in melanoma 2 protein like receptors
ALS	Amyotrophic lateral sclerosis
ApoE4	Apolipoprotein E
APP	Amyloid precursor protein
ARIA	Amyloid-related imaging abnormalities
ASC	Apoptosis-associated speck-like protein containing a CARD
Aβ	Amyloid β
CAA	Cerebral amyloid angiopathy
CARD	Caspase recruitment domain
CCF	Cytoplasmic chromatin fragments
CCL5	C-C motif chemokine ligand 5
CD200	Cluster of differentiation 200
CD68	Cluster of differentiation 68
CDR	Cytosolic DNA receptors
cGAMP	Cyclic GMP-AMP
cGAS	Cyclic GMP-AMP synthase
CLR	C-type lectin-like receptor
CNS	Central nervous system
CSF1R	Colony stimulating factor 1 receptor
CTT	C-terminal tail
CX_3_Cl1	C-X3-C motif chemokine ligand 1
CX_3_CR1	C-X3-C motif chemokine receptor 1
CXCl10	C-X-C motif chemokine ligand 10
DAI	DNA-dependent activator of IFN-regulatory factors
DAMP	Damage-associated molecular pattern
DAP12	DNAX activation protein of 12 kDa
DDX41	DEAD-box helicase 41
ERGIC	ER–Golgi intermediate compartment
FDA	Food and Drug Administration
FTD	Frontotemporal dementia
gDNA	Genomic-DNA
GWAS	Genome-wide association studies
HT	Huntington’s disease
IFI16	Interferon-gamma-induced protein 16
IFN	Type I interferon
IFNAR	Interferon-α receptor
IRF3	interferon regulatory factor 3
ISG	Interferon-stimulated genes
ITAM	Immunoreceptor tyrosine-based activation motif
LBD	Lligand-binding domain
LRR	Leucine-rich repeat
MAPK	Mitogen-activated protein kinase
MAPT	Microtubule-associated protein tau
MCI	Mild cognitive impairment
MEF2C	Myocyte enhancer factor 2c
mtDNA	Mitochondrial-DNA
MyD88	Myeloid differentiation primary response 88
NFT	Neurofibrillary tangles
NF-κB	Nuclear factor kappa B
NLR	Nucleotide-binding domain with leucine-rich repeat-containing receptors
NLRP	NLR containing a pyrin domain
NMDA	N-Methyl-D-aspartic acid
NSAID	Nonsteroidal anti-inflammatory drug
PAMP	Pathogen-associated molecular pattern
PD	Parkinson’s disease
PET	Positron emission tomography
PQBP1	Polyglutamine binding protein 1
PRR	Pattern recognition receptor
PYD	Pyrin domain
PYHIN	Pyrin and hematopoietic interferon-inducible nuclear family
RHIM	Receptor-interacting protein homotypic interaction motifs
RLR	Retinoic acid-inducible gene-like receptors
Stat1	Signal transducer and activator of transcription 1
STING	Stimulator of interferon genes protein
TBI	Traumatic brain injury
TBK1	Tank-binding kinase 1
TLR	Toll-like receptors
TREM2	Triggering receptors expressed on myeloid cells 2
ZBP1	Z-form DNA binding protein 1
